# Annotations

**Published:** 1896-01-18

**Authors:** 


### Annotations.

### A Latent Danger in Chlorosis.

Chlorosis is not usually classed among the more serious diseases, although probably its after-effects are sometimes graver than some people think. In comparison with its frequency dangerous complications are not very common, yet they do occur, and from their unexpectedness are all the more distressing. It is well, then, to draw attention to a dangerous complication which, however uncommon it may be, is so serious when it does take place as quite to throw into the shade all its contributory causes. We refer to thrombosis of the cerebral sinuses. At the last meeting of the Clinical Society, Dr. Lee Dickenson related a series of cases of apparently spontaneous thrombosis of the cerebral veins and sinuses in patients suffering from chlorosis, and from the discussion which followed it is clear that even fatal cases of this accident, if it may so be called, are by no means excessively rare, while cases which by good fortune escape the more serious issue may be far more common than some imagine. At any rate, where intense headache is associated with optic neuritis in the course of chlorosis, it is important not only to bear this condition in mind, but also not to forget that treatment is probably of great service in preventing its extension, even after it has begun, the free use of alcoholic and ammoniacal stimulants and alkalies, by which the activity of the circulation may be improved and the coagulability of the blood may be diminished, together with the administration of iron, being therapeutic means not to be neglected.

### The Fate of the Microbe.

We hear so much of microbes, and are so constantly assured that the air is full of them, that it becomes a matter of no small interest to ascertain how we are protected from them, or, in other words, how it is that, living as we do in the very midst of a cloud of micro-organisms, which we know by experience are able very quickly to reduce to putrescence substances which, so far as chemical composition goes, are like unto ourselves, we still remain protected from their attacks. The vulnerable point clearly is the mucous membrane of the air passages and the digestive organs. As regards the latter, we may well believe that in health we are protected by the activity of our digestive processes; but in reference to the air ducts, over the moist surfaces of which the foulest air is constantly drawn, it is a problem of the greatest interest to decide whereabouts the microbes, which we know are continually entering, are stopped. The recent researches of Dr. St. Clair Thomson and Dr. Hewlett, of the Bacteriological Department of the British Institute of Preventive Medicine, throw much light upon this question. They say that on an average about 1,500 micro-organisms are inhaled into the nose every hour; while in London it must be a common event for 14,000 of them to enter during one hour's tranquil respiration. Expired air, however, is practically sterile, and it would seem that this purification is not, as some have imagined, performed in the air-tubes of the lungs, for it has been found by repeated observation that they have vanished before reaching the trachea, the mucus from which is sterile. Evidently they are caught in the nose, for on testing air from the naso-pharynx they were found to be practically all gone. Nevertheless, the mucus in the nose does not appear to be itself a germicide. It does not kill the microbes, but it prevents their developing; and as microbes are only harmful by their monstrous power of multiplication this is sufficient. Meanwhile they are rapidly swept on by the cilia towards the digestive tract, where doubtless they share the common fate. The moral of all this is—breathe through your nose and keep your digestive organs in good working order, then the microbes, pathogenic, saprophytic, or whatever they may be, will meet their doom.

### Stuffy Railway Carriages.

An omnibus is a stuffy institution six on a side, and none too much room between the knees! But there are worse things than omnibuses. This popular mode of conveyance has at least an open door, a means of ventilation about six feet by two. Compare with that enormous opening the little holes over the top of the windows and doors of a railway carriage, when by the courtesy of one's fellow passengers even they are allowed to remain open, and judge then of the risks of railway travelling when the nights are cold and every intruder is—shall we say—blessed, not for his own sake, but because his opening of the door has let in the fog. We need not hesitate to say that, in winter time, the long railway journey which so many men undertake at each end of the day is not only a serious addition to the day's toil, but a definite addition to the day's danger. It is not a pleasant experience when a careful parent thinks he has got his family safely packed in a carriage, and fairly off to the seaside, to find a child enter whose neck is swathed in flannels and her ears stuffed with wool, and whose mother asks for the window to be put up, as her girl has "recently been ill." Unwelcome visions of diphtheria and scarlet fever may well crowd upon one's mind, and it becomes obvious enough that even the initial step in seeking health may involve considerable danger of contracting illness. Adults think themselves harder and imagine that they are free from the weakness of catching things, but it is only necessary to go back to the experience of a few years ago to assure oneself that this is not so, and that a good deal of the suburban influenza of those days was caught in trains and was conveyed into households by those whose daily work took them citywards by rail. It is difficult to see how this risk can be avoided, or even largely lessened. When it is remembered that eight passengers, and in second and third class carriages ten, are crowded into a space of not much more than four hundred cubic feet, a very slight consideration of the principles on which sanitarians calculate cubic space will show how seriously the air tends to be polluted before any long distance has been travelled. We do not wish to make too much of such matters. They are, to a large extent, inevitable, and form but a portion of that constant and intimate relationship between man and man, which, while the origin of many of his pleasures, is certainly the cause of many of his pains. Nevertheless, the risk of catching and importing infectious disease in the course of the daily journeyings to and fro should certainly be taken into consideration in deciding on the comparative advantages of living in town or country.

